# The effects of maternity waiting homes on the health workforce and maternal health service delivery in rural Zambia: a qualitative analysis

**DOI:** 10.1186/s12960-019-0436-7

**Published:** 2019-12-04

**Authors:** Jeanette L. Kaiser, Rachel M. Fong, Thandiwe Ngoma, Kathleen Lucile McGlasson, Godfrey Biemba, Davidson H. Hamer, Misheck Bwalya, Maynards Chasaya, Nancy A. Scott

**Affiliations:** 1 Department of Global Health, Boston University School of Public Health, Boston, MA USA; 2 Department of Research, Right to Care Zambia, Lusaka, Zambia; 3 National Health Research Authority, Pediatric Centre of Excellence, Lusaka, Zambia; 4 Section of Infectious Diseases, Department of Medicine, Boston Medical Center, Boston, MA USA

**Keywords:** Maternity waiting homes, Human resources for health, Skilled birth attendance, Postnatal care, Obstetric referrals, Rural health, Zambia

## Abstract

**Background:**

Maternity waiting homes (MWHs) are a potential strategy to address low facility delivery rates resulting from access-associated barriers in resource-limited settings. Within a cluster-randomized controlled trial testing a community-generated MWH model in rural Zambia, we qualitatively assessed how MWHs affect the health workforce and maternal health service delivery at their associated rural health centers.

**Methods:**

Four rounds of in-depth interviews with district health staff (*n* = 21) and health center staff (*n* = 73) were conducted at intervention and control sites over 24 months. We conducted a content analysis using a mixed inductive-deductive approach. Data were interpreted through the lens of the World Health Organzation Health Systems Framework.

**Results:**

Nearly all respondents expressed challenges with understaffing and overwork and reported that increasing numbers of facility-based deliveries driven by MWHs contributed substantively to their workload. Women waiting at MWHs allow staff to monitor a woman’s final stage of pregnancy and labor onset, detect complications earlier, and either more confidently manage those complications at the health center or refer to higher level care. District, intervention, and control site respondents passionately discussed this benefit over all time points, describing it as outweighing challenges of additional work associated with MWHs. Intervention site staff repeatedly discussed the benefit of MWHs in providing a space for postpartum women to wait after the first few hours of clinical observation through the first 48 h after delivery. Additionally, intervention site staff perceived the ability to observe women for longer before and after delivery allowed them to better anticipate and plan their own work, adjust their workloads and mindset accordingly, and provide better and more timely care. When understaffing and overwork were frequently discussed, this satisfaction in providing better care was a meaningful departure.

**Conclusions:**

MWHs may benefit staff at rural health centers and the health system more broadly, allowing for the provision of more timely and comprehensive obstetric care. We recommend future studies consider how MWHs impact the workforce, operations, and service delivery at their associated health facilities. Considering the limited numbers of skilled birth attendants available in rural Zambia, it is important to strategically select locations for new MWHs.

**Trial registration:**

Clinicaltrials.gov, NCT02620436. Registered December 3, 2015, https://clinicaltrials.gov/ct2/show/NCT02620436

## Background

The World Health Organization (WHO) recommends skilled care at every birth and postpartum care within 48 h of delivery to improve maternal and neonatal health outcomes [1, 2]. Increasing maternal health service utilization is needed to continue to improve maternal and neonatal health outcomes [1], which remain particularly low in rural areas of low-income countries [3–7]. However, many barriers persist that prevent women in these regions from delivering with a skilled provider or accessing timely postpartum care [5, 8, 9]. Maternity waiting homes (MWHs), temporary lodging for near-term pregnant women next to a health facility capable of providing obstetric care, have been proposed as a potential strategy to address access barriers to maternal health services experienced by women living in rural and resource-limited settings [10, 11]. These homes allow the most remote women, regardless of complication risk factors, to travel to a health facility in the weeks before their expected delivery date and reside there until labor begins, theoretically helping women to overcome the challenges of long distance and lack of transport. MWHs have existed for decades in various countries [11], and there is evidence they can increase access to health services, especially delivery with a skilled birth attendant, for the most rural women [12–15].

In Zambia, rates of facility delivery (2018: 83.8%) and postpartum care within 48 h (2018: 69.7%) have increased by 36.1 and 31.0 percentage points, respectively, over the last decade largely due to changes in government policy and large health systems interventions to promote both the supply of and demand for maternal health services [16–18]. However, improving rates mask the unequal distribution of service utilization within the country, and rural areas continue to lag behind. In 2018, the facility delivery rate in rural Zambia was 78.7%, compared to 93.2% in urban areas; the rate of postpartum care within 48 h of delivery was 63.6% and 81.5% in rural and urban areas, respectively [16]. Zambia’s maternal mortality rates, though decreasing, remain concerning with 252 maternal deaths per 100 000 live births [16]. To improve Zambia’s maternal health outcomes, rates of facility delivery and timely postpartum care must continue to improve, especially in the rural areas.

The majority of Zambia’s rural women access maternal health services at rural health centers, which provide primary care services and health education to a population between 5000 and 15 000 per site [19]. These centers often have low-quality infrastructure [19], with limited functional electricity [12]. Nearly all rural health centers provide continuous (24/7) service [12, 20], and basic maternal and child health services [19], with at least one skilled birth attendant on staff—usually a nurse or midwife [12, 19, 20]. There is variability in facility staffing [21], capacity to provide basic emergency obstetric and neonatal care (BEmONC) [12, 19, 22–24], and the ability to transport patients experiencing obstetric complications to referral centers [20].

Though MWHs are promising interventions to improve health service access for vulnerable populations, there is reasonable concern about increasing utilization at health centers in resource-limited settings which are already consistently overstretched, often with insufficient personnel skilled in birth attendance [25, 26]. Increased utilization of maternal health services could put an undue burden on an already taxed health system with overwhelmed health staff, potentially compromising the quality of care. Furthermore, sparse literature exists on how MWHs affect the health system and the ability of rural health center staff to provide care. While needing to understand the impact of MWHs on health outcomes is essential before implementing homes more broadly, understanding how functional MWHs affect the health system is also necessary.

Within a cluster-randomized controlled trial in Zambia, 10 community-designed and community-managed MWHs were constructed at rural health centers and evaluated against the standard of care for expectant women in four rural districts [27–29]. As part of the project’s implementation evaluation, a longitudinal qualitative study was conducted to assess how these new MWHs and the existing standard of care in rural Zambia affect the maternal health workforce and the service delivery to patients at their associated rural health centers.

## Methods

### Study setting

The MWH evaluation was conducted in rural health centers in Choma, Kalomo, and Pemba Districts in Southern Province, and Nyimba District in Eastern Province. The population of these districts is primarily rural, ranging from 69% in Choma/Pemba Districts (administratively combined during the 2010 census) to 91% in Kalomo and Nyimba Districts [30]. As of publication of The 2012 Health Facilities List in Zambia, Choma/Pemba Districts had 33 rural health centers serving an average 6650 people per facility, while Kalomo District had 31 rural health centers serving on average 8700 people each; Nyimba District had 17 rural health centers serving 6200 people each [19]. Each district has one or more hospitals (level 1 or level 2), which serve as the obstetric referral center for the district [19].

Between 2012 and 2016, these four districts, in addition to others in Zambia, received the Saving Mothers, Giving Life (SMGL) project, a multi-partner collaboration that took a holistic approach to address challenges around maternal and child health through a series of both supply- and demand-side interventions [18]. SMGL interventions targeted some aspects of the quality of care provided, including training and mentorship for health center staff in BEmONC, and improving electricity, water, and referral systems [22, 31].

### Intervention and standard of care description

Twenty rural health centers were selected from all eligible facilities in the study districts based on the following criteria: (1) distance to a referral facility (< 2 h), (2) capacity to perform five out of seven BEmONC signal functions [23, 24], and (3) volume of deliveries (> 150 per year) [29]. These selection criteria were employed to ensure the facilities were capable of providing basic obstetric care before any new MWHs were constructed, which hypothetically would increase delivery volume at the facilities. The rural health centers were pair-matched on delivery volume and distance to referral facilities, then randomly assigned to the intervention or control study arms. Full details of selection criteria and randomization procedures are available in the published study protocol [29].

Ten new MWHs were constructed according to community standards identified through a formative evaluation that showed community members sought MWHs that are comfortable, safe, culturally appropriate, and sustainable [27, 28, 32]. From this formative evaluation, a core MWH model was designed that included key domains for (1) infrastructure, equipment, and supplies to make the new homes comfortable, safe, and culturally appropriate; (2) policies, management structures, and financial systems to ensure the new homes are operationally and financially sustainable; and (3) health systems linkages and services to ensure women waiting at the MWH receive clinical services at the health center as well as health education [27, 29]. The MWHs are cement buildings with one large dormitory with beds, mattresses, and bedding for women awaiting delivery and one small dormitory for postpartum women, as recommended during formative research, for a total of 14 beds per facility. The homes also have latrines, private bathing and clothes washing areas, lockable cabinets for personal items, a cooking space with available pots and utensils, and a communal verandah for relaxation or health education classes. Nine of the ten new MWHs were opened in September/October 2016; one opened in March 2017.

The ten rural health centers randomized to the control group continued to operate under the “standard of care” for waiting women in the districts, which ranged significantly in quality [12]. Six sites had a community-constructed, one-room, mud-brick MWH where women slept on floors. In two sites, women slept on the health center ward floors (or beds if available) at night and waited outside during the day. One control site did not allow women to wait at the health center in preparation for or after delivery. One control site had women waiting in the wards until a new, quality MWH, similar in design to the infrastructure, equipment, and supplies domain of the core MWH model, was constructed during the course of the study.

### Data collection and management

A longitudinal qualitative evaluation was conducted with rural health center staff and district health officers to assess the effect of new and existing MWHs on the health system, which study staff hypothesized would change over time as utilization and staff responsibilities changed. We sought to capture nuanced changes by conducting four rounds of in-depth interviews (IDIs) at intervention and control sites over 24 months (November 2016 through October 2018), starting a few weeks after the first intervention sites opened. Approximately one staff member from each rural health center and one to two staff members from each district health office were interviewed during each round of data collection. Respondents were purposively sampled based on convenience—while we preferred to sample the health center in-charges, district health officer or staff directly involved in the MWHs at the health center or district levels, we were not always able to do this due to time constraints and availability of respondents. While some individuals may have been interviewed at more than one time point, most IDIs were conducted with different individuals due to changes in staffing, individuals being on leave or away for programs, and availability of individuals on the days of data collection.

Interview guides elicited information on the MWH strengths and challenges, their perceived impact on the health center workforce and service delivery, perceptions of MWH-associated costs, and sustainability of the MWH. Qualitative data collectors were trained in research ethics and interviewing techniques before each round of interviews. Informed consent and interviews were conducted in English, the common language of staff within the Zambian health system. Interviews lasted between 20 and 60 min and were conducted in a private space at either the health center or the district health office. Interviews were audio-recorded and transcribed verbatim into Microsoft® Word.

Demographic data were collected and entered into SurveyCTO Collect Software (Dobility Inc, Cambridge, MA) on tablets, then uploaded to a secure server only accessible by relevant project staff.

### Theoretical framework

**Fig. 1:**
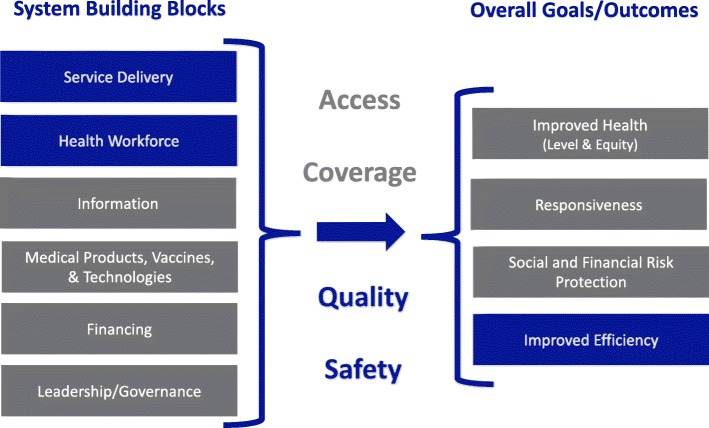
Theoretical framework used to guide the organization and interpretation of qualitative data. Adapted from the WHO [33]

We used the World Health Organization’s (WHO) Health System Framework, which defines six health systems building blocks and four overall goals, to organize and contextualize how health center staff and district health officers discussed the effect of the MWHs on the operations of rural health centers and the health workers’ perceived ability to provide maternity care to their patients (Fig. 1) [33]. Based on the characteristics of the intervention, we focused on the health workforce and service delivery building blocks, through the constructs of improved quality of care and safety, with the goal of improved efficiency of the health system on a microscale within each rural health center.

### Data analysis

IDIs were coded and analyzed in NVivo v11 (QSR International, Doncaster, Australia). The main codes were identified *a priori* based on the instrument guide, and new codes were added as themes emerged. Project staff conducted a content analysis using a mixed inductive-deductive approach [34, 35]. IDIs from each study arm were analyzed at each round and then compared over time and between study arms for each respondent type to identify patterns and key themes that were related to topics covered by the interview guide. Responses that were deviant from the observed patterns and themes were investigated further by study site to provide explanations, and the research team discussed their importance to the overarching findings. No deviant responses were considered sufficiently important for inclusion in the results below.

Demographic data were analyzed in SAS v9.4 (SAS Institute, Cary, NC). The clinical positions of the rural health center staff were collapsed into the following categories based on their international [1] or Zambia-specific [36] classification as a skilled birth attendant: (1) clinical officer, (2) nurse, (3) midwife, and (4) non-SBA staff (environmental health technologist, etc.). The district health staff positions were categorized based on oversight of the MWHs in the district: (1) district health officer, (2) maternal child health officer, (3) nursing officer, and (4) other officer. Some district-level respondents were “acting,” meaning they were standing in for another individual or had yet to be confirmed to their post. For the health center staff, 6.8% (*n* = 5) were missing employment information; length of employment was missing for one district health officer.

## Results

We first present the demographic information of the respondents, then present the major themes that emerged from the qualitative data regarding the MWH effect on health workers and service delivery. Illustrative quotes from the IDIs are included in tables and referred to throughout the results.

### Respondent demographics

Seventy-three (73) IDIs were conducted with rural health center staff, and 21 IDIs were conducted with district health officers over 24 months (Table 1). Respondents were equally split by sex, and the majority were the in-charges at their respective facilities. Approximately 54% of respondents were SBAs (clinical officers, nurses, or midwives); 27% were the in-charges of their facilities and therefore likely an SBA. Fewer respondents (33%) from the district health office were female. Approximately 14% of district respondents were the District Medical Officer, 33% were Maternal Child Health Officers, one was a Nursing Officer, and the remainder was a mix of officers of Environmental Health, Planning, Surveillance, and Public Health. On average, health center staff respondents had been working for about 10 years in their district, while district health officers had been working for slightly longer (12 years).

**Table 1:** Demographic characteristics of in-depth interview respondents by data collection round and overall

	R1	R2	R3	R4	Total
	Nov–Dec 2016	Apr–May 2017	Oct–Nov 2017	Jul–Oct 2018	
Rural health center staff	*N* = 21	*N* = 19	*N* = 15	*N* = 18	*N* = 73
Female, *N* (%)	11 (52.4)	11 (57.9)	6 (40.0)	6 (33.3)	34 (46.6)
Facility in-charge, *N* (%)*	14 (66.7)	10 (52.6)	3 (30.0)	10 (55.6)	37 (54.4)
Clinical position, *N* (%)*					
Clinical officer	2 (9.5)	1 (5.3)	2 (20.0)	1 (5.6)	6 (8.8)
Nurse	3 (14.3)	6 (31.6)	2 (20.0)	3 (16.7)	14 (20.6)
Midwife	7 (33.3)	3 (15.8)	2 (20.0)	5 (27.8)	17 (25.0)
Non-skilled birth attendant staff	4 (19.1)	3 (15.8)	3 (30.0)	3 (16.7)	13 (19.1)
In-charge, clinical position missing	5 (23.8)	6 (31.6)	1 (10.0)	6 (33.3)	18 (26.5)
Years working in the health system, mean (SD)*	10.7 (8.9)	10.2 (9.9)	6.9 (8.1)	11.6 (8.6)	10.2 (8.9)
District health officers	*N* = 6	*N* = 8	*N* = 3	*N* = 4	*N* = 21
Female, *N* (%)	2 (33.3)	3 (37.5)	1 (33.3)	1 (25.0)	7 (33.3)
Position, *N* (%)					
District Medical Officer	3 (50.0)	0 (0)	0 (0)	0 (0)	3 (14.3)
Maternal Child Health Officer	2 (33.3)	3 (37.5)	0 (0)	2 (50.0)	7 (33.3)
Nursing Officer	0 (0)	0 (0)	1 (33.3)	0 (0)	1 (4.8)
Other officer	1 (16.7)	5 (62.5)	1 (33.3)	2 (50.0)	10 (47.6)
Years working in the health system, mean (SD)^^^	11.4 (6.6)	11.4 (5.7)	8.7 (5.7)	16.2 (4.3)	11.9 (5.8)

*SD* standard deviation

*Missing 6.8% (*N* = 5) of data

^^^Missing 4.8% (*N* = 1) of data

### MWH effect on health workforce: increased responsibilities

IDI respondents discussed the effect of the MWH on the health workforce within the context of persistently understaffed health centers. Specifically, intervention and control site staff explained that while there may be two to three skilled birth attendants officially on staff, there is often only one available to manage deliveries at any given time because of leave and absences due to illness or trainings. Staff often reported managing more than one delivery concurrently or in close sequence, or being awoken for a delivery in the middle of the night while still needing to attend to work day responsibilities (see illustrative quotes 2a–2g in Table 2). Despite being overworked, staff often expressed trying their best, feeling a duty to their patients, repeatedly stating that the well-being of the mother and child is most important.

**Table 2:** Example quotes illustrating how health facility and district-level in-depth interview respondents discussed the challenges of understaffing and overwork at intervention and control sites

	Challenges of understaffing/overwork
Health facility staff respondents	
Intervention sites	2a. “We are already understaffed at this facility.…One person cannot do it all. Sometimes you find that maybe you delay here at the facility and the women at the MWH will say 'you have ignored us.'” (Health staff, intervention) 2b. “We are understaffed but we manage. So far there has never been a time when there is no one completely to attend to the mothers.” (Health staff, intervention site) 2c. “From the time the MWH was built, we ended up feeling that the number of staff that were there [at the health facility] was not enough, so we aired it out and the district officers started giving us more staff because sometimes we are encountering challenges of attending to people.” (Health staff, intervention site)
Control sites	2d. “Though we are understaffed we have to carry out these duties. I am the only midwife who has to run the labor ward as well as the maternal child health department, but we help our other colleagues. We do manage.” (Health staff, control site) 2e. “It’s always been a challenge in terms of workload.” (Health staff, control site) 2f. “Workload comes in because you have to see the people in the MWH, you have to see people in the maternal child health department, you have to see people at the outpatient department. We can’t leave the people alone in the MWH, they came here and they are in our hands.” (Health staff, control site)
District health staff respondents	
District health officers	2g. “The only bad aspect is we may need people to be full time with these mothers. Staffing is bringing a strain because if you have a mother in the ward, you are expecting to be checked and you find some facilities only have two staff to have a continuous check.” (District health staff)

Intervention and control site staff perceived the MWH as an extension of the rural health center and discussed similar responsibilities of staff toward the MWHs. In general, staff at rural health centers with associated MWHs are responsible for:

1. Monitoring waiting women by conducting clinical checks in the rural health center wards
2. Attending to deliveries
3. Educating waiting women on health topics
4. Cleaning and managing the MWH

District health officers and health center staff at both intervention and control sites all concurred that monitoring the health and wellbeing of the waiting mothers and their babies is the primary role of health center staff regarding the MWH. Health center staff reported conducting clinical checks on waiting women to identify any concerning antenatal or postpartum signs, and monitoring the onset of labor (see illustrative quotes 3a–3e in Table 3).

**Table 3:** Example quotes illustrating how in-depth interview respondents perceived the primary responsibilities of rural health center staff toward maternity waiting homes in intervention and control sites

	Primary responsibilities of health center staff toward the MWH			
	(1) Monitor waiting women	(2) Attend deliveries	(3) Educate waiting women	(4) Clean and manage MWH
Health center staff respondents				
Intervention sites	3a. “I think there is close monitoring of the mothers before they deliver. Mothers are being palpated twice in a week to detect if maybe there is a complication, which is then attended to in due time before anything happens.” (Health staff, intervention site) 3b. “All those women that are in the MWH are reviewed regularly and examined regularly to detect if there’s any problem with the women.” (Health staff, intervention site)	3f. “We have seen an increase in deliveries. Last month the MWH was very full, we had a lot of deliveries. Most of them were coming from the MWH to deliver.” (Health staff, intervention site) 3g. “People will come here two weeks or even a month before the expected date of delivery. So as a result, we don’t have home deliveries but health facility deliveries, and the numbers are increasing.” (Health staff, intervention site) 3h. To even double that number [of facility deliveries] to have just one midwife working was a challenge, because the midwife will be called every night and still need to work during the day.” (Health staff, intervention site)	3l. “When you look at the MWH, it is part of us as a facility, but we always find time to go and give health education to the mothers there.” (Health staff, intervention site)	3n. “We’re receiving a lot of mothers and then we have to make sure they’re well-kept and they are safe. It is our duty to see that everything is in order.” (Health staff, intervention site) 3o. “The health facility staff are responsible to ensure that the mothers who are at the MWH are safe and there are no conflicts. In the event where conflicts are reported, as a center, we have the responsibility to ensure that we resolve the conflicts amicably.” (Health staff, intervention site)
Control sites	3c. “Although there’s a lot of work overload, it’s very important that at least we monitor, we observe our mothers and the babies. It’s about their wellbeing.” (Health staff, control site) 3d. “It is good to have mothers waiting at the facility. We do check vitals twice a day. If we come across any problem then we refer there and then, unlike if they are at home, then it’s difficult.” (Health staff, control site)	3i. “It is a lot of pressure for the staff now because a lot of people are delivering at the facility, so the midwife tends to be overwhelmed.” (Health staff, control site) 3j. “The impact is that we have more women to deliver here hence there is a lot of work to do when the staffing still remains the same.” (Health staff, control site)	3m. “We also give health education on various topics like signs of labor, family planning, postnatal...Even for mothers who have delivered, we give health education on personal hygiene, family planning, the importance of breastfeeding and coming back for PNC.” (Health staff, control site)	3p. “In fact, we are the ones taking care of the structure. We make sure it is in good shape, it is well maintained. In terms of cleanliness, we need to make sure the surroundings are clean.” (Health staff, control site) 3q. “Some of our staff that clean the health facility, we do actually oblige them to also clean the MWH. Even sweeping around the place, it is done by staff.” (Health staff, control site)
District health staff respondents				
District health officers	3e. “We need to make sure that those mothers that make it to the MWH get the best attention - on a daily basis in terms of health facility staff checking on their baby, checking for danger signs, examining them.” (District health staff)	3k. “The positive impact is that a lot of women are now delivering at the health institution, but the challenge now is on the staff, because you find that the staff are overwhelmed. There is a lack of trained midwives at the facilities.” (District health staff )	No themes emerged	3r. “The role of the health center in-charge in terms of overall management of the MWHs is mitigated by the presence of those independent structures [community-derived committees], which are all linked to the general management of the health center, at least they are able to supervise and also to make sure some organizational arrangement is assured in the MWH.” (District health staff)

Health center staff are also responsible for attending to women in labor, regardless of whether the women utilized the MWHs. IDI respondents at the districts and health centers perceived increasing numbers of facility-based deliveries driven by the MWHs as contributing to staff workload (see illustrative quotes 3f–3l in Table 3). Intervention site staff reported this perceived increase in facility deliveries more often than control site staff and discussed increasing facility deliveries as challenging due to insufficient staffing more in later rounds of interviews (see illustrative quotes 3h–3i in Table 3). Similarly, district health officers recognized a need for greater human resources at the health centers, as the reported increase in deliveries at facilities with newly constructed MWHs became clearer (see illustrative quote 3l in Table 3). Some intervention sites were allocated additional staff from the district health offices in order to accommodate the additional volume.

At rural health centers, staff and volunteers from the community routinely conduct health education on personal hygiene, newborn danger signs, well-baby care, and family planning. Intervention and control site staff both discussed including waiting women at the MWHs in these health education classes (see illustrative quotes 3m–3n in Table 3).

The last major responsibility of health center staff toward the MWH is cleaning and managing the homes. While control staff discussed directly cleaning and managing the MWHs themselves, intervention site staff reported playing a supervisory role because the new homes were designed to be “community owned” with a committee of community members responsible for their daily operations (see illustrative quotes 3o–3r in Table 3). Some district health officers corroborated the importance of the community-derived MWH management structures at intervention sites, saying they mitigate the direct managerial role of the health center staff in the MWH operations (see illustrative quotes 3s in Table 3).

### MWH effect on service delivery: improved quality and safety

#### Labor monitoring and obstetric complication detection

Both health center staff and district health officers perceived that the benefits of the MWHs greatly outweighed the additional responsibilities previously discussed. Health center staff across all intervention and control sites frequently discussed the benefit of women arriving early to the health center because staff can monitor a woman’s final days or weeks of pregnancy, monitor the onset of labor, detect complications earlier, and either more confidently manage those complications at the health center or “refer in good time” and “only when necessary” to higher level care (see illustrative quotes 4a–4f in Table 4). Health center staff at both intervention and control sites passionately discussed this benefit over all time points. Most district health officers did not recognize the benefits of the MWHs for health center operations (separate from increased deliveries and access for remote women to services) until later rounds of interviews, after the new MWHs had been operating for approximately 12 months (see illustrative quotes 4g–4h in Table 4).

**Table 4:** Example quotes illustrating how in-depth interview respondents discussed the main benefits of maternity waiting homes on the health center staff workforce and maternal health service delivery at intervention and control sites

	Benefits of labor monitoring and obstetric complication detection	Benefits of postpartum observations	Benefits on work planning and job satisfaction
Health center staff respondents			
Intervention sites	4a. “Those women that are in the MWH are examined regularly so complications are detected early and referred in good time.” (Health staff, intervention site) 4b. “Complications are detected early and therefore referrals are made in good time. Before we had an MWH, complications were detected late and therefore, the prognosis and the outcomes of the deliveries were not good.” (Health staff, intervention site) 4c. “We are able to recognize the complications after delivery and able to refer to the hospital in time, unlike in the past (before the MWH).” (Health staff, intervention site) 4d. “The mothers have been coming earlier than when the labor starts. They are able to come in good time. Unlike in the past, where they would come maybe 30 minutes before the delivery time.” (Health staff, intervention site)	4i. “In terms of postnatal, at least we are able to see mothers for 48 hours. Before [the MWH], we discharged after they delivered, we were just able to observe them for six hours and then discharged them due to lack of space. But now, we are able to keep them. We take them to the MWH.” (Health staff, intervention site) 4j. “If they deliver today we keep the women for two days because we have the space there in the MWH. We do the postnatal at 48 hours then we discharge them. So even if we miss them at six days, we’ll have checked them at 48 hours, seeing the mother was okay and the child was okay.” (Health staff, intervention site) 4k. “The new MWH, with a capacity of four beds for postnatal mothers, is helping us to reduce on the congestion after delivery. We always have space.” (Health staff, intervention site)	4o. “The MWH is everything to the health facility staff. It brings a lot of easiness in going about our responsibilities. On one hand, workload has increased but on the other hand you get satisfaction and ensure that your obligations are fulfilled. We are doing our best to ensure balance.” (Health staff, intervention site) 4p. “The MWH has helped us as staff in providing the best service possible because we are able to make a quick decision on a problem as early as possible. We can only help someone properly if that person comes in at the right time to the clinic.” (Health staff, intervention site) 4q. “Each time when there are mothers there [at the MWH], we are always psychologically prepared to wake up at night. Compared to the way before [the MWH] when you go to sleep, and then after 10 minutes, someone comes saying, they have brought someone in labor.” (Health staff, intervention site) 4r. “In terms of work load, the MWH has actually made work easy for us, because we are able to identify the challenge ahead.” (Health staff, intervention site) 4s. “The MWH has actually made our work a bit lighter because we are able to do the correct things at the correct times.” (Health staff, intervention site)
Control sites	4e. “Our mothers will be near us as early as possible, so we will identify their problems early and then take a step. Those that we can’t handle, we’ll refer them early to the hospital.” (Health staff, control site) 4f. “It’s quite a lot of work. But then we are also looking at the wellbeing of a mother and the child. Some of them come from very far, they’ve got an opportunity to wait, and as they wait here we can also assess if they’ve got any danger signs. And even those who deliver, as least if a problem arises we are able to monitor it.” (Health staff, control site)	4l. “We can’t even see them at 48 hours because we have nowhere to keep them. After delivery, we’re supposed to keep a mother for 48 hours but we don’t have enough space. So for someone to come back from home after 48 hours, it’s not possible.” (Health staff, control site)	No themes emerged
District health staff respondents			
District health officers	4g. “For cases where our staff are able to monitor the patient who is in the MWH, it gives ample time for staff to actually make a decision. If it is an issue they know they are not able to handle, they are able to call for an ambulance way in advance. They are able to refer to the hospital.” (District health staff) 4h. “The facility staff are in contact with these mothers much earlier and they examine them, and those complications are being referred much earlier. There is an improvement in that assistance is given to the mothers early.” (District health staff)	4m. “There are about four bed spaces [in the intervention MWH sites] that once she delivers, the mother can wait there and do their first postnatal visit. We are already seeing those changes and we are seeing more mothers being able to access the first postnatal visit.” (District health staff) 4n. “In the past we didn’t have the capacity to keep a mother for 48 hours. The delivery room was small, the postnatal ward was small, and even the antenatal ward was small, so we couldn’t keep a mother after delivery, we were discharging after six hours, but this time we keep mothers up to 48 hours [at intervention MWH sites].” (District health staff)	No themes emerged

#### Postpartum observation and postnatal care attendance

Intervention site staff specifically and repeatedly discussed the important benefit of the MWH in providing a space for postpartum women and their newborns to wait after the first 6 h of clinical observation through the first 48 h after delivery (see illustrative quotes 4i–4k in Table 4). District officials expressed similar sentiments, anticipating this benefit in the first round and discussing it more during subsequent rounds of IDIs (see illustrative quotes 4m–4n in Table 4). Intervention site staff reported some women waiting up to six days at the MWH in order to attend their six-day postnatal care visit at the health center, rather than travelling the long distances to and from their homes. Health center staff expressed appreciation for the increase in postnatal attendance, allowing them to better monitor women during those critical postpartum hours and days. The intervention site staff reported feeling better about their ability to provide care for their patients and ensure women are in good health before returning home, unlike prior to the new MWHs when women would return home only 6 h postpartum due to lack of beds. The benefit of postpartum observation was nearly exclusively discussed at intervention sites, often attributed to the four-bed postnatal room included in each new MWH. Some control sites discussed their discontent with not being able to accommodate postpartum women at the health center for longer (see illustrative quote 4l in Table 4).

### MWH effect on health workforce: work planning and job satisfaction

Nearly all intervention site staff reported that the early arrival of women and retention of women for postnatal care allowed them to better plan their own work, anticipate better when they will be needed for a delivery, adjust their workloads and mindset accordingly, and provide care “at the right time” (see illustrative quotes 4o–4s in Table 4). For example, if a woman’s labor was progressing and she would likely deliver during the night, health center staff may rest earlier in the day to better care for her at night, instead of being awoken by a woman who has just arrived in labor. The intervention site staff were passionate about their ability to provide better care to antenatal, laboring, and postpartum women due to the MWHs, which made them feel better within their roles. When much of the IDI discussion regarding their role was about understaffing and overwork, this satisfaction in providing better care to their patients was a meaningful departure. This discussion of planning and satisfaction due to providing better care was rarely discussed at control sites.

## Discussion

To improve the health of its mothers and newborns and reach the goals set out in the 2017–2021 National Health Strategic Plan, including reducing maternal mortality to 162 deaths per 100 000 live births by 2021 [37], Zambia must continue to increase the availability of, quality of, access to, and utilization of maternal health services. MWHs have the potential to increase access to and utilization of skilled birth attendance [12–14, 38, 39], which remains lower in rural areas (73%) compared to urban areas (93%) of Zambia [16]. However, the added responsibility of overseeing waiting women could also create additional work for an already overburdened, resource-strapped health system [37], which exist in other rural sub-Saharan African settings [25, 26, 40–42]. This analysis sought, through the lens of the WHO Health System Framework, to understand the perceived effect new MWHs have on the health workforce and maternal health service delivery at rural health centers compared to facilities operating under the “standard of care” in rural Zambia. Since the majority of health center staff respondents are the in-charges of their facilities, are skilled birth attendants, and have worked in the health system of their district for over a decade on average, they have sufficient experience to comment on the effects of MWHs at their facilities. The district health officers provide additional insight and offer a higher level comparison between the intervention and control sites.

MWHs may benefit not only the women who utilize them, but also the health system more broadly through the delivery of maternal health services. Rural health center staff and district health officers in this study highlighted how MWHs provide space for women to wait for delivery, allowing health center staff to monitor women in the last weeks of pregnancy and early in labor, and to make timelier and more accurate diagnoses of antenatal complications (e.g., preeclampsia/eclampsia or placenta previa) and labor complications (e.g., prolonged stages of labor or fetal distress) for appropriate referral to higher-level care. As the outcome of such complications is often dependent on timely administration of appropriate medications or procedures, the time to referral and management of the complication is essential, especially in rural Zambia where distances between rural health centers and referral centers can be large and ambulances are rarely immediately available. Essentially, respondents perceive the MWHs allows the health workforce to provide better quality obstetric care to their patients by improving their workflow and increasing the efficiency of the obstetric referral system. Furthermore, MWH stays may allow for additional health system contact opportunities for women considered “high-risk” for complications [43], who may not have been identified during routine ANC visits. Understanding the role of MWHs from the perspective of the obstetric referral system can provide context to the findings of previous studies, such as a retrospective study of Ethiopian hospitals which found higher rates of caesarian sections and lower rates of maternal and perinatal mortality among MWH users compared to non-users [13].

Our results also suggest MWHs may offer an important opportunity to increase the low 48-h postnatal care coverage in rural areas (64%) [16], which is largely concentrated in the first 4 h after delivery (40%) [36]. Postnatal care is particularly important to identify and manage any potential postpartum complication, such as postpartum hemorrhage or puerperal sepsis, which may not arise for hours or days after birth and should be referred to higher-level care [43]. As intervention site staff attest, the new MWHs, with beds in a small postnatal-specific room, provide a comfortable and convenient space for women to wait after being released from clinical care for up to 48 h or even 6 days for their postnatal care visits. While intention of the MWH model was explicitly to *not* provide clinical care in the MWH space [27–29], it is clear that a benefit of the MWH from the perspectives of the providers is to offer additional, dedicated space for postpartum women to wait while they continue to be examined within the health center. Utilizing the MWH for postnatal stays in the absence of other suitable space, while technically still clinical care, has allowed facilities to retain women longer for postpartum observation.

While the MWH will inevitably generate additional work as evident from our findings, health center staff generally found the additional responsibilities minimally burdensome and regarded the MWH as a beneficial tool to better conduct their work. These individuals were genuinely committed to their work and to their patients, despite the broader, persistent challenge of feeling under-resourced and overworked, and explicitly perceived that an MWH allows them to better manage their patients who are awaiting delivery or are postpartum. Studies have shown that birth attendants are particularly vulnerable to burnout, at least partially due to workload and understaffing, and emotional exhaustion due to the nature of their jobs treating patients in acute pain and under stressful conditions [41, 42, 44, 45]. The health center staff at intervention sites in this study expressed feeling more comfortable with and more in control of their work due to the MWH because it allowed them to better anticipate when women would deliver so they could plan their schedules accordingly. This improvement in working conditions, especially for skilled birth attendants, may be motivating and could help retain these providers in rural health centers for longer, reducing burnout and turnover of staff, though this would require further study.

While multiple studies have assessed maternal and perinatal health outcomes in MWH-users compared to non-users [11, 13, 46], we recommend that future studies, in and outside of the sub-Saharan African context, also consider how the MWH impacts the operations of the health facility itself—be it a health center or hospital—regarding the workload of the health workforce, and the effect of the MWH on the management of complications or timely referral of patients to higher-level care. While the study described here utilized qualitative methods, we recommend future studies also employ quantitative methods to more systematically measure changes in health workforce job satisfaction and burnout, quality of obstetric services provided, and average time to referral for obstetric patients.

Within the context of increased deliveries due to MWHs, our results highlight the importance of considering the health center’s capacity when determining the placement of an MWH with the intention of generating demand. As Vermeiden and Stekelenburg have noted, MWHs should only exist at health facilities capable of providing BEmONC services and linked to higher-level care through a formal referral system [47]. It is, therefore, essential to only construct MWHs at health facilities with adequate and appropriately trained staff, medications, and equipment to manage the expected increase in delivery volume. While the pervasive human resource shortage continues to be a challenge across the Zambian health sector [37], district health staff seemed to use MWHs and their perceived benefits to leverage additional human resources for specific clinics. It is, therefore, necessary to engage with district health officers responsible for human resource distribution within their districts to appropriately site new MWHs and to advocate for additional staff at facilities with new MWHs. Without sufficient numbers of skilled birth attendants available to conduct deliveries, it is possible that increasing numbers of deliveries could compromise the quality of care provided, decrease staff morale, and undermine the community’s confidence in that health center.

## Limitations

There are two main limitations with this analysis. First, this is a purely qualitative analysis and perceptions of trends or increased delivery are not independently validated. However, the perception of increased demand is important in understanding the impact felt by the health center staff and the exploration of perceptions of multiple stakeholders across multiple time points strengthens the findings.

Second, not all rural health centers or district health officers were available for interviews each round. Every effort was made to reach each site each round, but some sites could not be included every round due to logistical constraints and the availability of the potential respondents. Due to the large volume of qualitative data, we do not believe that this biases the data as a missed site would be included in the following round.

## Conclusion

In the context of a shortage of human resources for health in rural Zambia, although health center staff perceive an extra workload associated with MWHs, they strongly believe that MWHs help them to deliver better maternal health services, including more timely management of obstetric complications and more appropriate referrals. These trends are particularly important to consider in light of Zambia’s commitment to increasing access to maternal health services and reducing maternal mortality.

## Data Availability

The in-depth interview transcripts included in this analysis are not publicly available due to ethical restrictions to publicly sharing data which are of sensitive nature and contain potentially identifiable information. Data requests may be sent to the Boston University IRB at medirb@bu.edu. Demographic data are available from the corresponding author on reasonable request.

## Abbreviations

BEmONC: Basic Emergency Obstetric and Neonatal Care
IDI: In-depth interview
IRB: Institutional Review Board
MWH: Maternity waiting home
SMGL: Saving Mothers, Giving Life Project
WHO: World Health Organization
